# Glypre: In Silico Prediction of Protein Glycation Sites by Fusing Multiple Features and Support Vector Machine

**DOI:** 10.3390/molecules22111891

**Published:** 2017-11-03

**Authors:** Xiaowei Zhao, Xiaosa Zhao, Lingling Bao, Yonggang Zhang, Jiangyan Dai, Minghao Yin

**Affiliations:** 1 School of Computer Science and Information Technology, Northeast Normal University, Changchun 130117, China; zhaoxw303@nenu.edu.cn (X.Z.); zhaoxw303@126.com (X.Z.); baoll601@nenu.edu.cn (L.B.); 2 Key Laboratory of Symbolic Computation and Knowledge Engineering of Ministry of Education, Jilin University, Changchun 130012, China; zhangygcq@163.com; 3 School of Computer Engineering, Weifang University, Weifang 261061, China; longwind111@126.com

**Keywords:** glycation sites, support vector machine, feature analysis

## Abstract

Glycation is a non-enzymatic process occurring inside or outside the host body by attaching a sugar molecule to a protein or lipid molecule. It is an important form of post-translational modification (PTM), which impairs the function and changes the characteristics of the proteins so that the identification of the glycation sites may provide some useful guidelines to understand various biological functions of proteins. In this study, we proposed an accurate prediction tool, named Glypre, for lysine glycation. Firstly, we used multiple informative features to encode the peptides. These features included the position scoring function, secondary structure, AAindex, and the composition of *k*-spaced amino acid pairs. Secondly, the distribution of distinctive features of the residues surrounding the glycation and non-glycation sites was statistically analysed. Thirdly, based on the distribution of these features, we developed a new predictor by using different optimal window sizes for different properties and a two-step feature selection method, which utilized the maximum relevance minimum redundancy method followed by a greedy feature selection procedure. The performance of Glypre was measured with a sensitivity of 57.47%, a specificity of 90.78%, an accuracy of 79.68%, area under the receiver-operating characteristic (ROC) curve (AUC) of 0.86, and a Matthews’s correlation coefficient (MCC) of 0.52 by 10-fold cross-validation. The detailed analysis results showed that our predictor may play a complementary role to other existing methods for identifying protein lysine glycation. The source code and datasets of the Glypre are available in the Supplementary File.

## 1. Introduction

In general, glycation is a post-translational modification produced by a reaction between reducing sugars and the amino groups of lysine or arginine, or N-terminal amino acids. It is a two-step non-enzymatic reaction. The first step is to form the stable Amadori product based on the unstable Schiff base. The second step is to further form irreversible cross-linked products, the so-called advanced glycation end products (AGES). The accumulation of glycation products are known to associate with the pathogenesis of aging and complications of diabetes. It also plays crucial regulatory roles in almost all cellular processes and is involved in other human diseases, such as Alzheimer’s [1] and Parkinson’s diseases [2]. The essence of glycation is the reducing sugars attached to amino groups in cellular proteins, which result in Schiff bases as early glycation products [3,4]. Recently, more interest has been paid to lysine glycation from researchers working on metabolism [5].

Although glycation has been found relevant with an increasing number of cellular process, the systematic identification of glycation sites is still challenging since the glycated residues do not show significant patterns. The conventional experimental techniques, such as CHIP-CHIP analysis and mass spectrometry are usually time-consuming, laborious, and expensive to detect glycation [6]. Thus, the computational approaches which could effectively and accurately identify the glycation sites are urgently needed. Recently, several computational approaches with machine learning approaches to predict modification (PTM) sites have been reported. Wei et al. [7] showed a novel sequence-based predictor for phosphorylation sites, which sufficiently explored the sequential information from multiple perspectives. Chen et al. [8] used an ensemble of support vector machines combining SVM-PseKNC, SVM-motif, and GkmSVM for detecting *N*^6^-methyladenosine sites from RNA transcriptomes. Jia et al. [9] established a high-accuracy predictor for protein dephosphorylation sites, which applied the sequence-based bi-profile Bayes feature extraction technique to identify three phosphatases and selected the weight parameters of the support vector machine (SVM) according to jackknife cross-validation. Zhao et al. [10] fused different features for phosphothreonine sites. Until now, only three computational methods have been developed to identify the glycation sites. Johansen et al. [11] developed the first glycation predictor, GlyNN, by combining 60 artificial neural networks with a balloting procedure. Liu et al. [12] established an improved predictor called “PreGly”. PreGly utilized the maximum relevance minimum redundancy method (mRMR) followed by the incremental feature selection produce (IFS) to reduce feature dimensions based on a support vector machine. Xu et al. [13] explored the application of sequence order information and position-specific amino acid propensity in glycation prediction problem, and provided a larger training dataset to train more reliable models called “Gly-PseAAC“. Although these predictors have been developed for the prediction of glycation sites, some problems still need to be taken into consideration. First, with more and more glycation sites being experimentally verified, it is necessary to establish a novel improved prediction model. Second, the biological hallmarks around the glycation sites have not been systematically investigated. Third, the accuracy of prediction is still not satisfied, so that there is still room to improve the performance.

**Figure 1:**
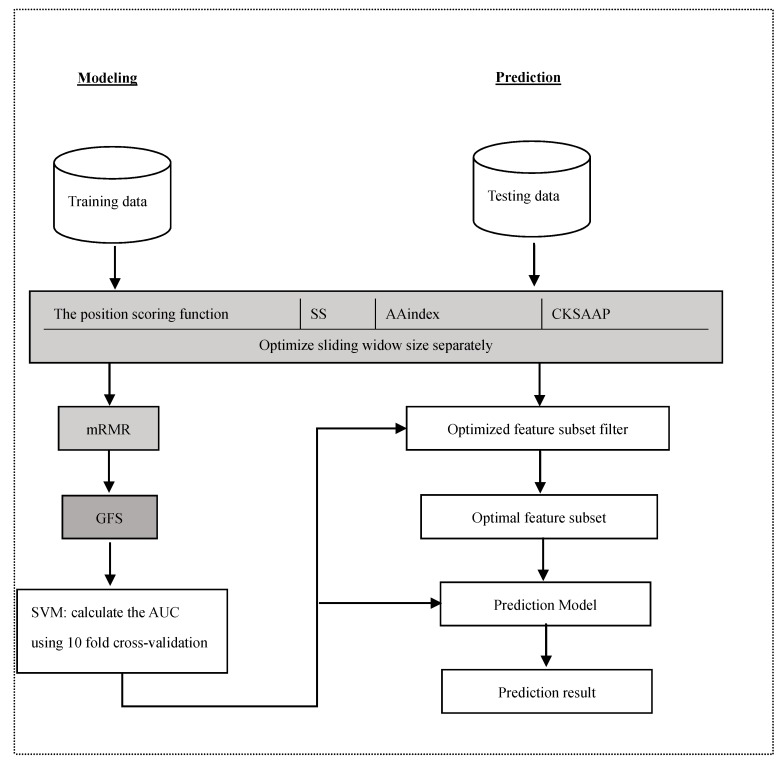
System architectures of the proposed method.

Thus, this study used a large training dataset [13] to train the reliable models and statistically analysed the distribution of properties. We also explored the application of some features in the lysine glycation prediction problem, and used a novel two-step feature selection, which was the maximum relevance minimum redundancy method followed by the greedy feature selection procedure (GFS), to remove the redundancy and contradiction among features to improve the prediction and generalizability of the model. The new predictor of identifying lysine glycation sites is called Glypre. In this study, a number of discriminative features, including the position scoring function, secondary structure (SS), AAindex, and the composition of *k*-spaced amino acid pairs, were constructed to encode the proteins. These informative features have been proved to be associated with PTM site prediction. We statistically found that the distribution of various properties of residues are perilously different between glycation and non-glycation peptides. Feature analysis also revealed that the encoding was efficient to capture a glycation site’s characteristics. Additionally, based on the distribution of feature properties, we used different optimal window sizes. The detailed analysis results in this study may provide useful information to detect lysine glycation sites. The framework of the proposed method is shown in Figure 1.

## 2. Results and Discussion

### 2.1. Investigation of Different Features

The residue distribution surrounding lysine is an important factor for the lysine glycation sites. Thus, for Dataset 1, the investigation is performed for the distribution of different properties, including position conservation, secondary structure, AAindex, and the composition of *k*-spaced amino acid pairs on the basis of a window size 31.

### 2.2. The Position Conservation Features Analysis

**Figure 2:**
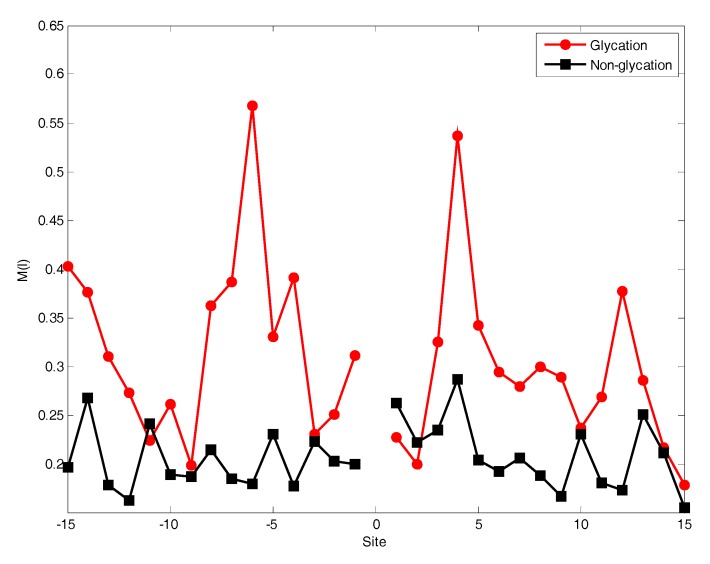
The position conservation *M(l)* value around the glycation and non-glycation sites.

The position conservation of residues surrounding the lysine can usually provide much helpful information to predict glycation sites. The analysis was performed for the distribution of the *M(l)* value of each position around lysine residues, as shown in Figure 2.

**Figure 3:**
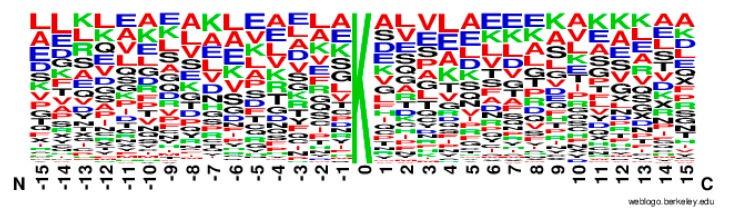
The sequence logo of lysine glycation sites.

From Figure 2, it can be observed that the *M(l)* value of almost each position in positive samples is greater than that in negative samples. It makes clear that these glycation sites prefer some special residues. Especially, it was found that the *M(l)* value at −6 and +4 sites were significantly higher than other sites, indicating that these two sites play relatively more important roles for glycation. Further analysis shows that the frequency of alanine (A), glutamic acid (E) and leucine (L) is >10% at −6 and +4 sites of glycation. From the sequence logo of the experimental 210 glycation peptides (Figure 3), we also found that alanine (A), glutamic acid (E), leucine (L), and lysine (K) have preferences to appear near the glycation sites. The results also support that acidic amino acids, mainly glutamate (E), and lysine (K) residues, catalyse the glycation of nearby lysines [11,14,15]. All in all, these analyses suggested that position conservation influenced the glycation.

### 2.3. Secondary Structure Features Analysis

**Figure 4:**
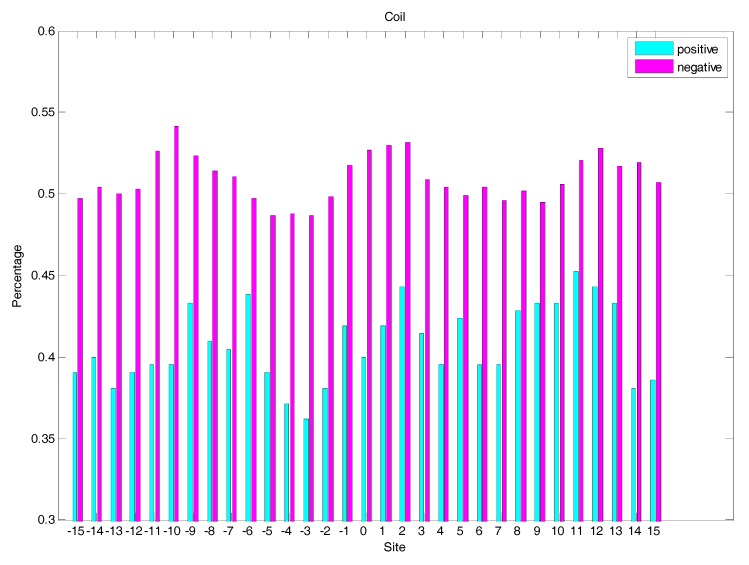
The distribution of coil structures of residues around glycation and non-glycation sites.

**Figure 5:**
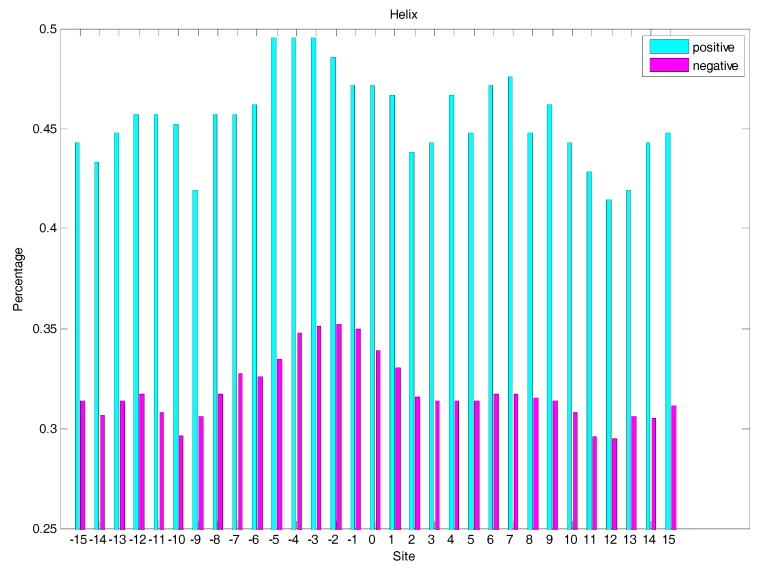
The distribution of helix structures of residues around glycation and non-glycation sites.

**Figure 6:**
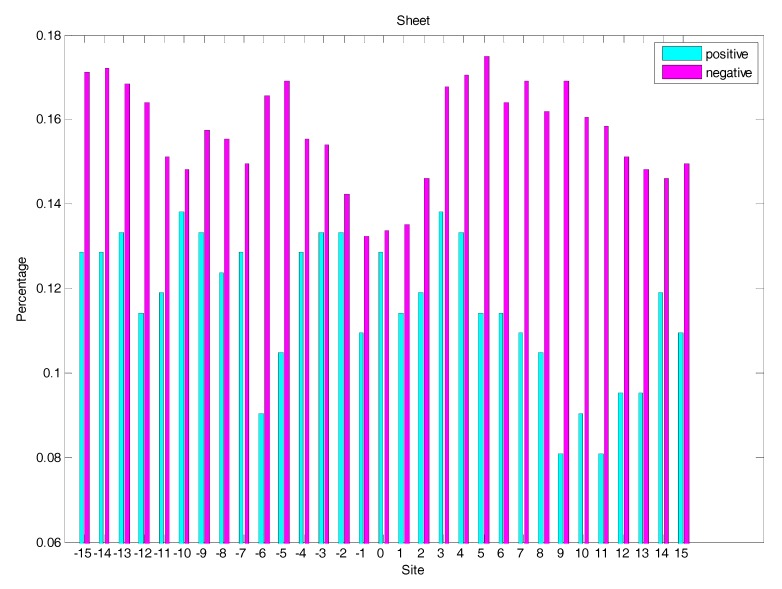
The distribution of the sheet structure of residues around glycation and non-glycation sites.

In order to analyse the difference of secondary structure (SS) between glycated and non-glycated peptides, the distribution of SS around lysine residues is calculated, as illustrated in Figure 4, Figure 5 and Figure 6, which demonstrates that the residues around the glycation site favoured to form helix structures. Moreover, it was observed that the closer the site gets to the glycation sites, the lower the frequency of helix and sheet structures, and the greater the frequency of coil structures, whereas the value of coils and sheets surrounding non-glycation sites were higher than that around the glycation sites. Moreover, the frequency of helix and sheet structures has a similar fluctuation in positive and negative samples. We can conclude from the above that the difference of SS could discriminate glycation and non-glycation sites efficiently.

### 2.4. AAindex Features Analysis

**Figure 7:**
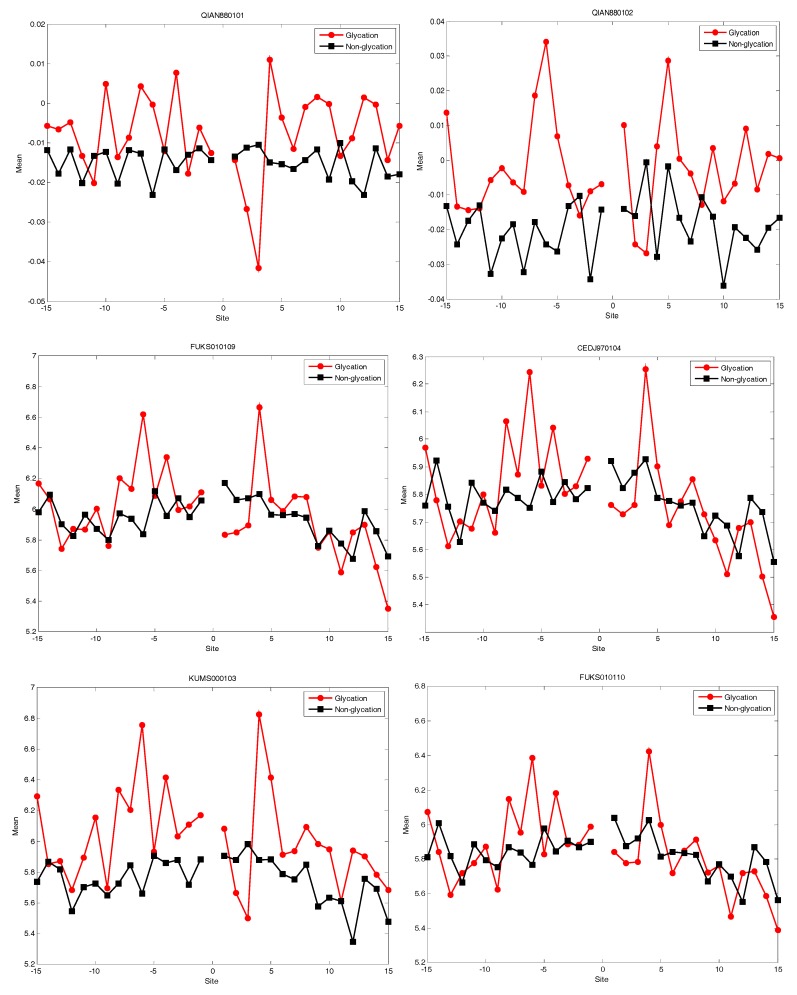
The distribution of the six amino acid indices of residues around glycation and non-glycation sites.

We knew that The Amino Acid Index database (AAindex) played very important roles in PTM prediction [16]. In this study, we selected the top 20 amino acid indices (shown in Table 1) corresponded to the best AUC value. Afterwards, the distribution of five of the 20 amino acid indices of residues around the glycation and non-glycation sites was statistically analysed. The six representative amino acid indices were shown in Figure 7. More details for the distribution of 20 amino acid indices are shown in the Supplementary Information. The results show that, for the 20 amino acid indices, the positive samples have a larger fluctuation than the negative samples. It also illustrated that information about the 20 amino acid indices around glycation and non-glycation sites was very different.

### 2.5. CKSAAP Feature Analysis

**Figure 8:**
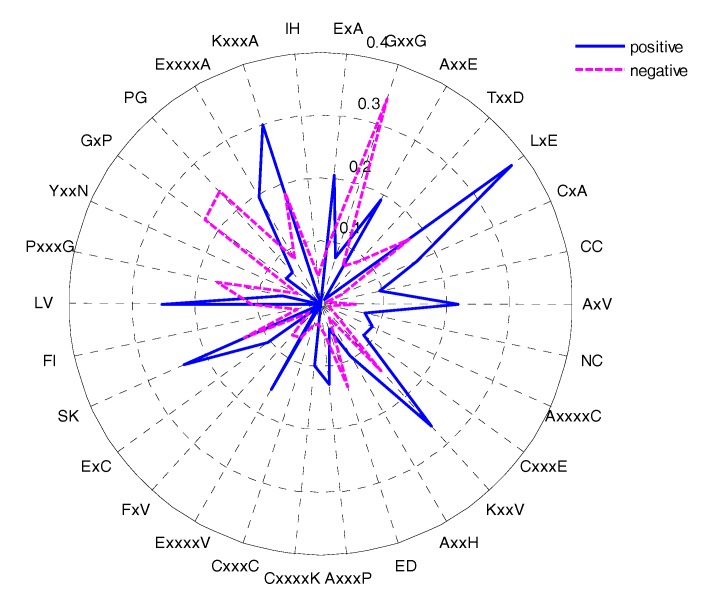
The composition of the top-30 residue pairs resulting from the IG method.

**Figure 9:**
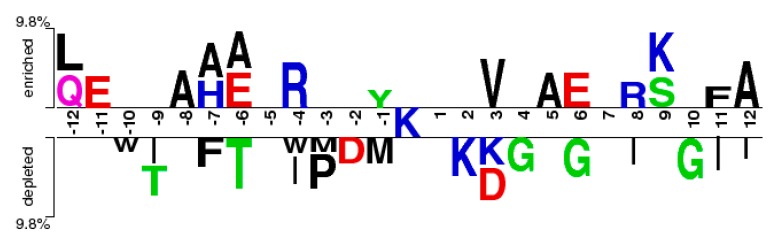
The two-sample-logos of the position-specific residue composition surrounding the glycation and non-glycation sites.

The feature selection method could determine the most important amino acid pairs, which were generated by the CKSAAP encoding scheme [17]. The CKSAAP is dependent on the window size, so we selected the optimal window size (17) to analyse the CKSAAP. In order to give some instruments for predicting the glycation sites, the top 30 features were selected according to the IG feature list. The composition of the top-30 residue pairs were also presented in two radar diagrams (Figure 8). As can be seen from Figure 8, the compositions of the top-30 features are remarkably different in glycation and non-glycation sites. The importance of the top-30 residue pairs is also clearly and intuitively characterized in Figure 9. For example, the feature ‘LxE’ is significantly enriched in position pairs (−12/−11) surrounding the glycation sites. As can be seen in Figure 9 A, E, and L frequently appeared in the top-30 amino acid pairs, which is consistent with the observation from Figure 8. Figure 9 also showed the sequence patterns around the glycation sites, that is, a sequence fragment including these amino acid pairs would more likely have glycation sites. Figure 8 also illustrated that information about the composition of the top-30 residue pairs was very different in positive and negative samples.

### 2.6. The Performance of the Proposed Predictor

According to the above analysis, we respectively optimize the window size of each set of features to obtain the best prediction accuracy. The window size is varied from 11 residues to 31 residues and investigated the AUC found through SVM in 10-fold cross-validation. The optimal window sizes are 31, 27, 13 and 25 residues, respectively. Based on the optimal window sizes for four groups of features, the sample can be formulated as a 402-dimension vector, including 31 dimensions for the position scoring function, 27 × 3 = 81 dimensions for the secondary structure, 13 × 20 dimensions for the AAindex, and 30 dimensions for the CKSAAP.

**Figure 10:**
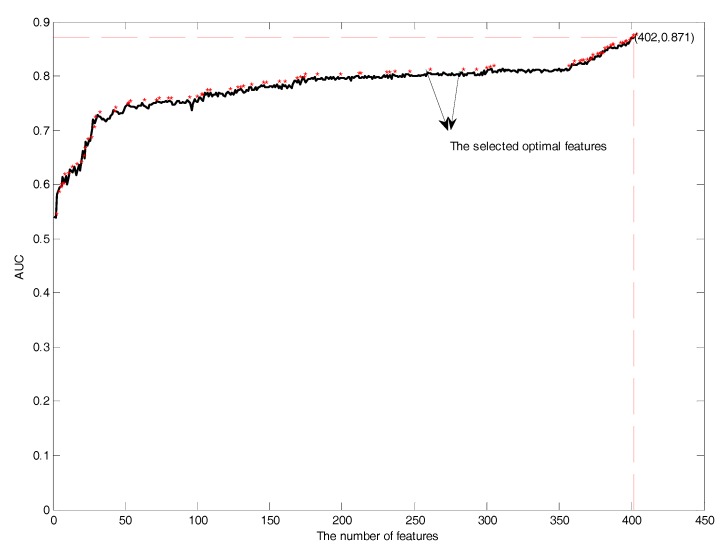
The GFS curves of glycation site prediction.

Next, it is necessary to perform feature selection to remove the irrelevant and redundant features. In this study, features in the mRMR feature rank list were added one by one during the GFS procedure by using SVM in 10-fold cross-validation. Performance comparisons of the prediction models with the addition of features is shown in Figure 10. The red asterisk showed the distribution of the selected optimal features. The number of selected optimal features was 87, which had the highest AUC of 0.87. The selected optimal features included the position scoring function (17), secondary structure (21), AAindex (32), and the composition of *k*-spaced amino acid pairs (17).

In order to evaluate the classification model in broader terms, the *k*-fold cross-validation test (*k* = 6, 8 and 10) is used by splitting the dataset into *k* equally-sized subsets, and using each of the *k* subsets as the testing dataset iteratively. The *k*-fold cross-validation test was executed 50 times with different random seeds to extract most representative statistical results. Meanwhile, we used the leave-one-out test as the classification evaluation strategy to avoid the sampling bias of the *k*-fold cross-validation test. The performance of the proposed method was shown in Table 2. In can be observed that Glypre performs statistically well for the sensitivity, specificity, accuracy, AUC, and MCC. Additionally, based on the same benchmark Dataset 1, our Glypre yielded a MCC of 0.52, a specificity of 90%, an accuracy of 79%, and an AUC of 0.86, which were observably better than an MCC of 0.31, a specificity of 54%, an accuracy of 68%, and an AUC of 0.72 in the Gly-PseAAC [13]. The sensitivity of 57.62% with Glypre was slightly worse than the sensitivity of 58.74% with Gly-PseAAC.

### 2.7. Comparison with Existing Methods

To ensure a fair comparison with previous studies, including Gly-PseAAC, GlyNN, and PreGly, Dataset 2 (89 glycation and 126 non-glycation sequences of 20 proteins) was adopted to investigate Glypre in three- and 10-fold cross-validation.

Table 3 shows the results of comparison. Glypre outputs the average values 20 times and other results of Gly-PseAAC, GlyNN, and PreGly have been cited from a paper [13]. Glypre achieved the highest Sen of 85.11%, Acc of 89.77%, AUC of 95.57, and MCC of 78.84, which was clearly better than the existing methods. Although the Spe of Glypre is less than the Sp of PreGly, the difference is not large.

### 2.8. Comparison with Other Predictors on the Independent Test Dataset

Since we have already investigated our proposed algorithm by *k*-fold cross-validation, it is necessary to further narrow down the independent dataset test to check the effectiveness of the final model. We randomly selected 20 proteins from PLMD 3.0, which had no overlap with Dataset 1. The performance of model on Dataset 1 was evaluated using the 20 proteins as independent testing dataset. A glycation prediction tool (Gly-PseAAC) still provided online prediction services (http://app.aporc.org/Gly-PseAAC/).

The performance of the proposed method was shown in Table 4. From Table 4, we can see that Glypre can successfully detect the same number of glycation sites as the Gly-PseAAC does. However, the threshold θ of Gly-PseAAC was set to 0.35, while the threshold θ of Glypre was set to 0.5. We can also see that the posterior probability scores of Glypre were almost larger than that of Gly-PseAAC. This means that Glypre is a promising method for the prediction of protein glycation sites.

## 3. Materials and Methods

### 3.1. Datasets

The experimental-confirmed lysine glycation benchmark dataset (Dataset 1) used in this work was collected from the database CPLM (http://cplm.biocuckoo.org/) [18]. There were 323 lysine glycation sites extracted from 72 proteins, and the corresponding primary protein sequences were collected from UniProt (release 2014_11, http://www.uniprot.org/) [19]. Redundant sequences were removed by using the CD-HIT [20] program with at least a 30% pairwise sequence identity threshold. Finally, a total of 47 proteins which contain lysine glycation sites were obtained. Subsequently, from these proteins, 210 peptides having length of 31 residues and experimental-validated glycation in the centre were collected and labelled them as positive samples. Correspondingly, the other 31-mer sequences with lysine centres, which are non-glycation sites, were selected as the negative samples. Finally, there were 210 positive samples and 1383 negative samples. The number of the positive and negative samples is unbalanced which usually results in a skewed classification of non-glycation. Thus, a main dataset was obtained by combining the 210 positive samples and 420 negative samples randomly selected from the 1383 negative samples. Although Gly-PseAAC [13] used the same benchmark dataset, it only provided the samples which are 15 residues long with the lysine in the centre. Thus, the comparison with the Gly-PseAAC on the benchmark dataset is not so propitious.

To evaluate the effectiveness of the proposed method as well as to perform fair comparisons with previous methods [11,12,13], we used another benchmark dataset (Dataset 2) to train Glypre, which contained only 89 glycation sites and 126 non-glycation sites from 20 proteins. This dataset can be downloaded from http://www.cds.dtu.dk/databases/GlycateBase-1.0/.

To further test the generalizability of our method, an independent testing dataset was introduced in this work which was derived from the database PLMD 3.0 (http://plmd.biocuckoo.org/) [21]. We randomly selected the 20 proteins from PLMD 3.0, which had no overlap with Dataset 1. These 20 proteins including 37 lysine glycation sites were constructed as the independent testing dataset.

### 3.2. Feature Construction

#### 3.2.1. The Position Scoring Function

The potential sequence characteristic of residues around glycation sites (from −15 to +15) is one of the most important aspects. Here, we investigated the residue preference at each site by the following conservation formulation (Equation (1)):

(1) $$M(l)=\sum_{i}^{20}\frac{{\left(P_{i}^{l}-p_{0}\right)}^{2}}{p_{0}}$$

where $P_{i}^{l}$ indicates the occurrence frequency of the *i*th amino acid at the *l*th position. *p*_0_, which represents the background frequency, is set to 0.05. The larger the conservation of the *M(l)* value is, the stronger the conservation of the *l*th site. When the *M(l)* value is 0, it represents a random distribution of the 20 residues at the *l*th position.

Given the aligned training sequences from positive samples, the position weight matrix (PWM) was defined as follows Equation (2):

(2) $$P_{xl}=\frac{n_{xl}+p_{0}\sqrt{N}}{N+\sqrt{N}}$$

where *n_xl_* denotes the real counts of residue *x* at the *l*th position. *p*_0_ is the background frequency of each amino acid in the protein sequence, and equal to 0.05 in this work. *N* denotes the number of the training sequences. Then, with the arbitrary peptide fragment, which has 31 residues, the position scoring function [10] of the *l*th site can be calculated as Equation (3):

(3) $$F(l)=\ln\frac{P_{xl}}{p_{0}}\times [M_{p}(l)-M_{N}(l)]$$

where *M_p_*(*l*) and *M_N_*(*l*) denote the position conservation at the *l*th site in the positive and negative samples, respectively. The value of *F(l)* shows the degree of the sequence close to positive samples.

#### 3.2.2. The Secondary Structure (SS)

In this step, the secondary structure information around glycation sites is taken into account, which plays a vital role in the protein’s structure and function [22]. Secondary structures include α-helix, β-sheets, and coil. PSIPRED [23] is taken into account for the prediction of secondary structures, which delivered the output in the form of “H”, “C”, and “E”, representing helix, sheets, and coils, respectively. In order to constitute a numeric vector, we used the probability of “H”, “C”, and “E” to encode protein segments in this study.

#### 3.2.3. Amino Acid Indices

The Amino Acid Index database (AAindex) includes amino acid mutation matrices and amino acid indices [24]. It collected 544 physicochemical properties for version 9.0. An amino acid index including 20 numerical values stands for physicochemical properties of 20 amino acids. Physicochemical properties have been successfully predicted for several protein modifications [25,26]. Here, we selected 20 informative physicochemical properties to encode each peptide in this work.

#### 3.2.4. The Composition of *k*-Spaced Amino Acid Pairs (CKSAAP)

The composition of *k*-spaced amino acid pairs (CKSAAP) has been successfully used for predicting various PTMs [12,17,27], which could reflect the characteristics of residues surrounding glycation sites. Given 20 native amino acids and one complementary residue ‘X’, the CKSAAP feature contains 441 basic amino acid pair types: AA, AC, …, AW, AY, XX. The basic amino acid pair types are enlarged to the *k*-spaced amino acid pair types. For example, ‘A^W’ means that this amino acid pair is separated by one other amino acid. Considering that the CKSAAP was performed over *k* = 0, 1, 2, 3 and 4 in this study, and the vector size of the CKSAAP feature was 2205 dimensions, here, we utilized the information gain method (IG) [28] to rank the 2205 features. Afterwards, the top-30 features to encode the protein were selected.

### 3.3. Feature Selection

Feature selection is an important step for building an effective prediction model [29,30,31,32,33]. Generally, not all features have an equivalent contribution to the glycation prediction system. In addition, some features are usually noisy and redundant. To analyse the features, we used the mRMR method [34] to rank all the features. Then, we implemented a new GFS procedure based on the mRMR rank list. The GFS procedure is as follows: Features in the mRMR feature rank list were added one by one to make the feature subset, and if the AUC of the feature subset fulfils the criteria of improvement by means of an SVM in 10-fold cross-validation, and this feature is added. Finally, the feature subset that has the highest AUC was used to train the model for predicting glycation sites.

### 3.4. Support Vector Machine

The support vector machine (SVM) derives from statistical learning theory, first proposed by Vapnik [35], which is an effective machine learning technique. SVM constructs a hyperplane that separates two types of samples as widely as possible in a high-dimensional space. It has been effectively applied in many bioinformatics problems. In this study, we utilized the LIBSVM toolset [36]. A grid search strategy based on 10-fold cross-validation is utilized to find the optimal parameters.

### 3.5. Performance Assessment

Five measurements were employed to evaluate the performance of our proposed predictor. These measurements included sensitivity (SN), specificity (SP), accuracy (ACC), and Matthews’s correlation coefficient (MCC), and area under the receiver-operating characteristic (ROC) curve (AUC). AUC is the area under the receiver-operating characteristic (ROC) curve, presented as a plot of true positive rate against false positive rate. The AUC score of a ROC curve summarizes the overall performance of a corresponding model or method [37], while others are defined by the following formulas Equations (4)–(7):

(4) $$Sn=\frac{TP}{TP+FN}$$

(5) $$Sp=\frac{TN}{TN+FP}$$

(6) $$Ac=\frac{TP+TN}{TP+TN+FP+FN}$$

(7) $$MCC=\frac{TP\times TN-FP\times FN}{\sqrt{\left(TP+FN)\times (TN+FP)\times (TP+FP)\times (TN+FN\right)}}$$

where *TP*, *TN*, *FP*, and *FN* represent the number of true positives, true negatives, false positives, and false negatives, respectively.

## 4. Conclusions

In this work, we explored the application of a position scoring function and secondary structure (SS) in the glycation prediction problem. The distribution of different properties around the glycation sites and non-glycation sites based on a large training dataset were statistically analysed. The analysis suggests that the proximity of acidic amino acids, mainly glutamate, and lysine to lysines promotes the glycation. We also found some important features which can contribute to the prediction of glycation. A novel two-step feature selection was proposed, which improved the prediction and generalization ability of the model. The experimental results of Glypre outperformed the existing methods on Dataset 2, which revealed the effectiveness of this new method. Moreover, the promising performance on an independent testing dataset from the PLMD database also proved the commendable generalization ability of the defined method. In the future, we will collect more data, analyze more features, and use other useful strategies to construct a predictive model with higher accuracy. Of course, the method forged in this research can also be used in the prediction of other protein post-translational modifications.

## Figures and Tables

**Table 1:** The accession numbers of the 20 amino acid indices.

Accession Number			
QIAN880101	FUKS010109	RACS820107	QIAN880118
FUKS010102	CEDJ970104	NAKH920106	GEIM800107
FUKS010101	KUMS000103	KARP850103	PARS000102
QIAN880102	CHAM830102	FUKS010104	FUKS010110
PALJ810108	RACS820104	QIAN880104	BURA740102

**Table 2:** Experimental results for our proposed predictor on Dataset 1. The results are the mean values (standard variation).

Cross-Validation	Sen (%)	Spe (%)	Acc (%)	AUC	MCC
10-fold	57.47 (1.31)	90.78 (0.56)	79.68 (0.57)	0.8629 (0.0035)	0.5232 (0.0140)
8-fold	57.10 (1.41)	90.95 (0.65)	79.67 (0.71)	0.8629 (0.0050)	0.5227 (0.0175)
6-fold	56.30 (1.74)	91.06 (0.74)	79.47 (0.88)	0.8600 (0.0059)	0.5175 (0.0218)
LOO	57.62	90.24	79.37	0.8693	0.5162

**Table 3:** Experimental results for Glypre and the existing methods Gly-PseAAC, GlyNN, and PreGly on Dataset 2.

Predictor	Sen (%)	Spe (%)	Acc (%)	AUC	MCC
Glypre ^a^	85.11	93.06	89.77	0.9557	0.7884
Glypre ^b^	80.96	91.55	87.16	94.20	0.7344
Gly-PseAAC	56.06	80.17	68.12	0.7705	0.38
PreGly ^a^	71.06	95.85	85.51	-	0.70
GlyNN ^b^	78.65	80.15	79.50	0.77	0.58

^a^ The result was obtained by 10-fold cross-validation ^b^ the result was obtained by three-fold cross-validation.

**Table 4:** Experimental results for Glypre and Gly-PseAAC on the independent test dataset. We highlighted the posterior probability scores of successfully detecting glycation sites. The glycation sites of protein were listed, and the posterior probability scores of these two predictors were also shown.

Protein	Glycation	Glypre	Gly-PseAAC
P62760	7, 18	0.3278, 0.5980	<0.35, 0.3835
Q9Y5I3	677	0.8407	0.5878
Q9Y6P5	55	0.2178	<0.35
A6NE02	302	0.0722	<0.35
Q9NPC3	119	0.7346	0.3831
P29122	573	0.4627	<0.35
O96005	207, 209	0.3228, 0.3646	0.5519, 0.5515
P47869	231, 247	0.7250, 0.2822	<0.35, <0.35
Q8TC59	770	0.2745	<0.35
Q8IUR6	216, 493	0.1153, 0.2307	<0.35, <0.35
Q9Y587	53	0.0524	<0.35
P28289	191, 214, 221, 228, 249, 255, 286, 297, 308, 314	0.4737, 0.1668, 0.3140, 0.4933, 0.1197, 0.4824, 0.1432, 0.1932, 0.0227, 0.2218	<0.35, <0.35, 0.7305, 0.4924, <0.35, <0.35, <0.35, <0.35, 0.3583, <0.35
O94919	252, 281, 300	0.0252, 0.4890, 0.6587	<0.35, <0.35, 0.3520
P01877	155	0.1260	<0.35
Q93034	137	0.2791	<0.35
Q13011	267, 276	0.6926, 0.8448	<0.35, 0.4122
Q6P6C2	274	0.0244	<0.35
Q8IZI9	70	0.5000	<0.35
Q15084	73, 245	0.6981, 0.0404	<0.35, <0.35
Q8IY21	1077	0.5758	<0.35

‘<0.35’ indicates the posterior probability score is less than 0.35.

## Supplementary Materials

Supplementary materials are available online.
